# Farnesoid X Receptor Agonists as Therapeutic Target for Cardiometabolic Diseases

**DOI:** 10.3389/fphar.2020.01247

**Published:** 2020-08-26

**Authors:** Chao Li, Jie Yang, Yu Wang, Yingzi Qi, Wenqing Yang, Yunlun Li

**Affiliations:** ^1^Experimental Center, Shandong University of Traditional Chinese Medicine, Jinan, China; ^2^Cardiovascular Department, Affiliated Hospital of Shandong University of Traditional Chinese Medicine, Jinan, China; ^3^School of Health, Shandong University of Traditional Chinese Medicine, Jinan, China

**Keywords:** Farnesoid X Receptor, cardiometabolic diseases, lipid metabolism, diabetes mellitus, obesity

## Abstract

Cardiometabolic diseases are characterized as a combination of multiple risk factors for cardiovascular disease (CVD) and metabolic diseases including diabetes mellitus and dyslipidemia. Cardiometabolic diseases are closely associated with cell glucose and lipid metabolism, inflammatory response and mitochondrial function. Farnesoid X Receptor (FXR), a metabolic nuclear receptor, are found to be activated by primary BAs such as chenodeoxycholic acid (CDCA), cholic acid (CA) and synthetic agonists such as obeticholic acid (OCA). FXR plays crucial roles in regulating cholesterol homeostasis, lipid metabolism, glucose metabolism, and intestinal microorganism. Recently, emerging evidence suggests that FXR agonists are functional for metabolic syndrome and cardiovascular diseases and are considered as a potential therapeutic agent. This review will discuss the pathological mechanism of cardiometabolic disease and reviews the potential mechanisms of FXR agonists in the treatment of cardiometabolic disease.

## Introduction

Cardiometabolic diseases are reaching epidemic proportions in the world and the leading cause of death in both developed and developing countries (Matsuzawa et al., 2011). Cardiometabolic diseases are characterized by a combination of multiple risk factors for cardiovascular disease (CVD) and metabolic diseases including type 2 diabetes (T2D), obesity and dyslipidemia (Arnett et al., 2019; Ralston and Nugent, 2019). Individuals with cardiometabolic diseases face a higher risk of developing myocardial infarction, stroke and heart failure (Landsberg et al., 2013). According to a May 2017 report of the World Health Organization, CVD are the leading cause of death worldwide, with 17.9 million people dying from CVD in 2016, representing 31% of all global deaths. Cardiometabolic diseases are linked to several risk factors, in particular high-salt and high-fat diet (HFD), smoking, hypertension, and high cholesterol. Mounting evidence-based medical data indicates that hypercholesterolemia is an independent risk factor for CVD (Birbeck et al., 2015; Wald et al., 2016). Reducing abnormally high levels of cholesterol can effectively decrease the occurrence of cardiovascular events. Over the past few decades, the obesity rate and prevalence of T2D have increased significantly worldwide (Lascar et al., 2018; Bluher, 2019). The increase in the prevalence of diabetes and obesity has further contributed to the increase in the incidence rate of cardiometabolic diseases and continues to undermine the management of CVD (Zhang et al., 2018). In addition, a major cause of increased morbidity and mortality of cardiometabolic diseases is the increasing population of aging. Projections indicate that the prevalence of CVD in the United States may escalate by 10% between 2010 and 2030. The estimated increase stems in part from the aging of the population (Vasan and Benjamin, 2016). Measures to achieve healthy aging and alleviate aging-related morbidity have become a burning issue for health care (Ren et al., 2018). Understanding mechanism of aging is of immense clinical importance to population-wide cardiovascular risk. Although statins and angiotensin converting enzyme inhibitors improve cardiovascular outcomes, there is still a percentage of patients with cardiometabolic diseases still face the risk of developing overt cardiovascular disease.

Farnesoid X receptor (FXR) is a member of the nuclear receptor superfamily, and was identified as a receptor of bile acids (BAs) (Li C. et al., 2019). There is one human gene for FXR (*NR1H4*) separately from the rodent genes (*Nr1h4* and *Nr1h5*), and human *NR1H5P* is a pseudogene. The human and rodent *Nr1h4* genes are conserved and encode four different protein isoforms (FXR*α*1, FXR*α*2, FXR*α*3, and FXR*α*4), which are produced by alternative promoters and RNA splicing (Teodoro et al., 2011). FXR is mainly expressed in various tissues, including the liver, intestine, and kidney. In recent years, FXR has been found to also be expressed in cells of the cardiovascular system, such as cardiomyocytes and endothelial cells. Besides being a bile acid receptor, FXR is also a bile acid synthesis biosensor, FXR participates in the synthesis, conjugation, absorption and secretion of BAs (Tu et al., 2000). FXR has also been shown to have a regulatory effect on glucose and lipid metabolism, intestinal flora metabolism, oxidative stress, and inflammation (Li H. et al., 2019; Schoeler and Caesar, 2019). Therefore, increasingly more attention has been paid lately to the therapeutic role of FXR in cardiovascular physiology and pathology. Moreover, FXR also showed effective therapeutic effect on nonalcoholic fatty liver disease (NAFLD) and metabolic diseases including obesity, diabetes, and hypercholesterolemia (Wang et al., 2018). In this review, we will focus on the potential mechanisms of FXR agonists in the treatment of cardiometabolic disease.

## FXR Modulators

As other nuclear receptors, the FXR protein also exhibits a highly-conserved DNA binding domain (DBD) to bind to the ligand (Modica et al., 2010). BAs are a common agonist, including primary BAs, such as chenodeoxycholic acid (CDCA), and cholic acid (CA), and secondary BAs, such as lithocholic acid (LCA), and deoxycholic acid (DCA). Several non-BA agonists of FXR have also been discovered, and according to their chemical structure are classified as steroidal or non-steroidal (Carotti et al., 2014). Several FXR agonists have been investigated in phase II and phase III clinical trials, and some candidate compounds have also been found to regulate FXR activity in pre-clinical studies (**Table 1**).

**Table 1:** Bile acid agonists and its derivatives.

Name	Chemical structures	Preclinical studies	Current clinical status	References
Obeticholic acid (OCA, INT-747)	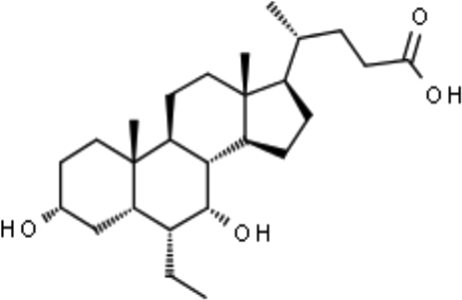	NASH mice; estrogen-induced cholestasis; rodent models of cholestasis.	Phase II for Lipodystrophy (NCT02430077); Phase IV for Primary Biliary Cholangitis (NCT02308111)	(Fiorucci et al., 2005; Carino et al., 2020; Li et al., 2020)
INT-767	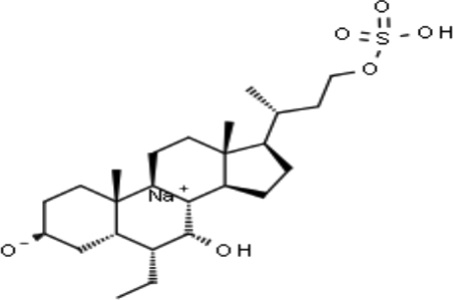	Chronic cholangiopathy (Mdr2^-/-^ Abcb4^-/-^ mice); *Lep*^ob/ob^ mice with NASH; HFD induced metabolic disorders.	NA	(Baghdasaryan et al., 2011; Jadhav et al., 2018; Roth et al., 2018)
TC-100	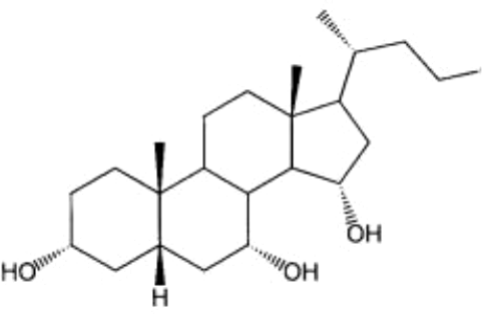	Bile fistula rats and bile duct ligation mice	NA	(Pellicciari et al., 2016)
GW4064	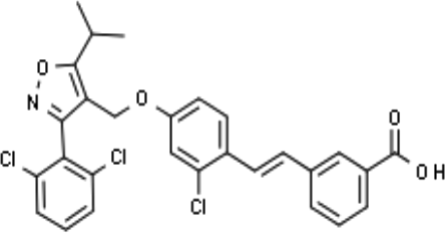	Short bowel resection rats associated with liver disease; endotoxin-induced hepatic inflammation; diet-induced obesity and hepatic steatosis.	NA	(Ma et al., 2013; Yao et al., 2014; Cao et al., 2019)
Px-102 (PX20606) and its eutomer Px-104 (GS-9674)	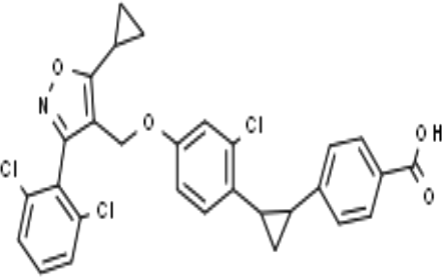	non-cirrhotic and cirrhotic portal hypertension; High-density lipoprotein-mediated transhepatic cholesterol efflux in mice.	Phase II for NAFLD (NCT01999101, completed). No current study	(Hambruch et al., 2012; Schwabl et al., 2017)
TERN-101 (LY2562175)	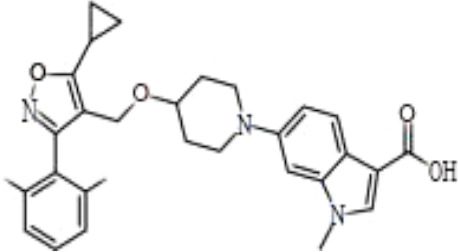	Dyslipidemia (LDLr-/- mice)	Phase II for NASH (NCT04328077)	(Genin et al., 2015)
Tropifexor (LJN452)	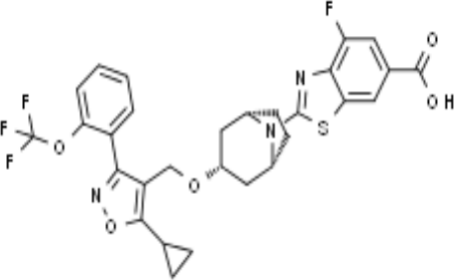	Amylin liver NASH model	Phase II for NAFLD and NASH (NCT03517540, NCT04147195)	(Hernandez et al., 2019)
WAY-362450	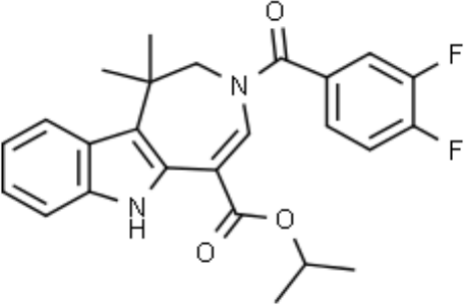	Maternal cholestasis; fructose-induced hepatic steatosis; murine model of alcoholic liver disease; murine model of non-alcoholic steatohepatitis.	Phase I (NCT00499629, completed; NCT00509756, terminated). No current study	(Zhang et al., 2009b; Liu et al., 2014; Wu et al., 2014; Wu et al., 2015)
Fexaramine	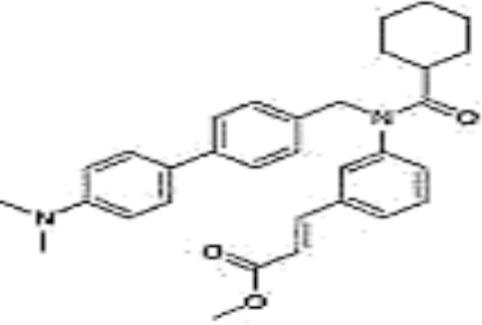	Alcoholic liver disease in mice; obese and diabetic mice, *Lep*^ob/ob^ mice; obesity and metabolic syndrome.	NA	(Fang et al., 2015; Hartmann et al., 2018; Pathak et al., 2018)
LMB763	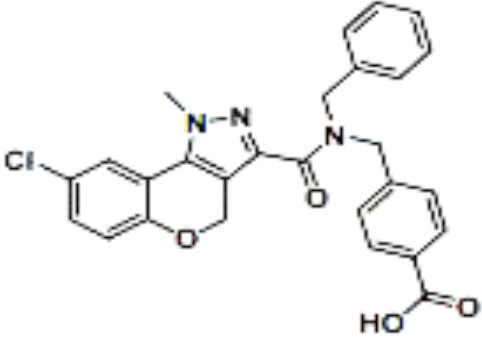	STAM and NASH murine mode	Phase II for Diabetic Nephropathy (NCT03804879)	(Chianelli et al., 2020)
EYP001	unknown	NA	Phase II for NASH (NCT03812029); Phase II for Chronic Hepatitis B (NCT04365933)	NA
EDP-305	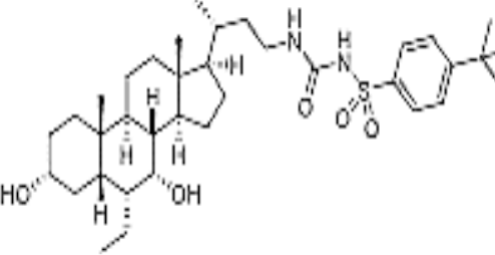	Biliary fibrosis and steatohepatitis mice; unilateral ureteral obstruction mice; choline-deficient, L-amino acid-defined, high-fat diet models of hepatic injury.	Phase II for Primary Biliary Cholangitis (NCT03394924); Phase II for NASH (NCT04378010)	(Erstad et al., 2018; Li S. et al., 2019; An et al., 2020)

NA, not available; NASH, non-alcoholic steatohepatitis; NAFLD, Nonalcoholic fatty liver disease; HFD, High-fat diet.

### Bile Acid Agonists and Its Analogues

#### Natural BAs and OCA

BAs are considered as important endogenous FXR agonists in different tissues. The potency of natural BAs to activate FXR follows the order CDCA>DCA>LCA>CA (Wang et al., 2018). Some studies have found that replacing the carboxylic group on the side chain of CDCA with 6α-alkyl significantly enhances its activating potency and efficacy on FXR, which is consistent with the hydrophobic nature of the pocket in FXR that is complementary to the 6α position (Pellicciari et al., 2006). The first FXR agonist that was investigated in clinical practice was obeticholic acid (OCA), which has an ethyl group substituted at the 6 position of CDCA, which is the most potent natural ligand activator of FXR (Pellicciari et al., 2002). OCA was approved by the United States (US) Food and Drug Administration for the treatment of primary biliary cholangitis (PBC) and evaluated in additional clinical trials for non-alcoholic steatohepatitis (NASH) (NCT01265498), alcoholic hepatitis (NCT02039219) and lipodystrophy (NCT02430077). However, besides its obvious therapeutic effect, OCA also has side effects, such as pruritus, and may participate in the regulation of blood lipids (Hirschfield et al., 2015; Neuschwander-Tetri et al., 2015). Future studies are needed to explore the effects of OCA on serum lipoprotein metabolism, which may be associated with CVD.

#### INT-767

INT-767, a semisynthetic BA that acts as a dual FXR and TGR5 (G-protein coupled receptor) agonist, has been found to be a slightly more potent activator of FXR, which also has the ability to alleviate liver injury and metabolic disorders (Baghdasaryan et al., 2011). It has been reported that INT-767 improves the NASH histopathological characteristics in a diet-induced *ob/ob* mouse model, showing greater efficacy than the treatment with OCA (Roth et al., 2018). Also, INT-767 has been shown to alleviate hypercholesterolemia and increase the expression of thermogenic genes through FXR and/or TGR5 activation, leading to the reversal of HFD-induced metabolic disorders (Jadhav et al., 2018). Moreover, since TGR5 is an important regulator of metabolism and energy homeostasis, INT-767 shows a broader and more effective therapeutic potency.

#### TC-100

TC-100 (3α,7α,11β-trihydroxy-6α-ethyl-5β-cholan-24-oic acid) is the first semisynthetic BA that combines the ability to specifically bind and activate FXR without TGR5 activation (Pellicciari et al., 2016). The study of the activation of FXR by TC-100 by cell-based analysis revealed that TC-100 is slightly more potent than OCA, and is highly effective in increasing the clearance of BAs from the liver to the bile canaliculus (Pellicciari et al., 2016).

Additionally, several other BA derivatives, including NIHS700 (Suzuki et al., 2008), 22(R)-OHC [oxysterol 22(R)-hydroxycholesterol] (Deng et al., 2006), androsterone [5α-androstan-3α-ol-17-one] (Wang et al., 2006), MFA-1 [17β-(4-hydroxybenzoyl) androsta-3,5-diene-3-carboxylic acid] (Soisson et al., 2008), have also been identified as FXR agonist.

### Non-Bile Acid Agonists

#### GW4064 and Its Derivatives

GW4064 is an agonist of FXR that belongs to a class of trisubstituted isoxazole core compounds with high affinity for FXR, which was reported in 2000 (GERARDUS et al.). GW4064 was reported to reverse BA dysmetabolism and alleviate hepatotoxicity in rats with short bowel resection associated with liver disease (Cao et al., 2019). GW4064 also attenuated lipopolysaccharide (LPS)-induced hepatic inflammation (Yao et al., 2014) and diet-induced hepatic steatosis and insulin resistance (Ma et al., 2013). However, GW4064 could only serve as a tool compound due to its poor bioavailability (Akwabi-Ameyaw et al., 2008) and potential hepatobiliary toxicity (Bass et al., 2011). A further study found that the chemical structure of GW4064 contains a stilbene olefin, which may be responsible for its poor bioavailability and photolabile properties. Px-102 (PX20606) and its eutomer Px-104 (GS-9674) are highly effective GW4064 derivatives, in which the stilbene olefin is replaced by a cyclopropyl moiety, with high affinity for FXR, which improved the aqueous solubility and membrane permeability. A phase I study with Px-102 was completed in healthy volunteers in 2011 and 2012 (NCT01998672, NCT01998659). Other studies have reported that Px-102 showed therapeutic potentials in the treatment of other diseases, including BA synthesis dysfunction (Al-Khaifi et al., 2018), vascular remodeling and sinusoidal dysfunction (Schwabl et al., 2017).

Px-104 (GS-9674, also called cilofexor) was considered as the best example of FXR agonist in the clinical development derivative. A single 100 -mg dose of cilofexor showed good tolerance in healthy volunteers and no grade 3 adverse effects were observed (Nelson et al., 2017). A Phase II trial with Px-104 (NCT01999101) was also conducted to evaluate the safety and tolerability of Px-104 in NAFLD patients. A Phase II randomized controlled trial (NCT02854605) with cilofexor in patients with NASH reported that cilofexor for 24 weeks was well tolerated and significantly reduced hepatic steatosis, liver biochemistry, and serum BAs (Patel et al., 2020). Another randomized, placebo-controlled trial (NCT02943460) also suggested that cilofexor significantly improved liver biochemistry and markers of cholestasis in patients with primary sclerosing cholangitis (Trauner et al., 2019).

#### LY2562175 and LJN452

LY2562175, a compound developed by Eli Lilly and Company, was evaluated in a European Phase I trial and subsequently licensed to Terns Pharmaceuticals and renamed TERN-101 (Genin et al., 2015). LJN452 (also called tropifexor) is a powerful and highly effective FXR agonist *in vivo*, which introduces a bicyclic nortropine-substituted benzothiazole carboxylic acid moiety into a trisubstituted isoxazole scaffold building on the chemistry structure of the Eli Lilly and Company compound LY2562175 compound (Tully et al., 2017). In healthy volunteers, tropifexor was well tolerated, had a pharmacokinetic curve indicating it was suitable for once daily administration and showed dose-dependent target binding without changing plasma lipids (Badman et al., 2020). Preclinical evaluation in animal models has demonstrated that tropifexor is a promising investigational therapy and it is currently under phase II (CLJC242A2201J; NCT03517540) development for NASH and liver fibrosis (Hernandez et al., 2019; Pedrosa et al., 2020).

#### WAY-362450

WAY-362450 is a highly selective FXR agonist [EC(50)= 4nM, Eff=149%] without cross-reactivity with other nuclear receptors at concentrations up to 10 μM (Flatt et al., 2009). WAY-362450 was reported to decrease LPS-induced serum amyloid P component and have anti-inflammatory effects (Zhang et al., 2009a). Several animal studies have demonstrated that WAY-362450 has effective therapeutic effect on atherosclerotic lesion formation (Hartman et al., 2009), hepatic fibrosis and NASH (Zhang et al., 2009b), and regulation of triglyceride and cholesterol homeostasis (Evans et al., 2009). However, WAY-362450 exhibits insufficient aqueous solubility due to its planar structure and lack of free ionizable group (Wang et al., 2018).

#### Fexaramine

Fexaramine is one of the earliest synthetic nonsteroidal FXR agonists with a 100-fold increased affinity relative to natural compounds (Downes et al., 2003). Fexaramine has become the prototype of the intestine-restricted FXR agonist, and has been shown to elicit potent beneficial metabolic effects without the side effects due to the activation of liver FXR (Fang et al., 2015). In addition, it has also been found that fexaramine has effective therapeutic effect on stabilizing the gut barrier and modulating hepatic lipid metabolism (Hartmann et al., 2018). Further studies have shown that fexaramine improves lipid profiles, increases glucose and insulin tolerance, and promotes browning of adipose tissue (Fang et al., 2015; Pathak et al., 2018).

#### Others

LMB763 (nidufexor), a novel non-BA FXR agonist, is based on a tricyclic dihydrochromenopyrazole core, which has partial FXR agonistic activity *in vitro* and FXR-dependent gene modulation *in vivo* (Chianelli et al., 2020). LMB763 has advanced to Phase II clinical trials in patients with NASH and diabetic nephropathy. EYP001 is a synthetic FXR agonist developed by ENYO Pharma and currently in Phase II trial for hepatitis B virus (HBV) infection as well as for NASH treatment. However, the chemical structure of EYP001 is unknown and there are no peer-reviewed publications on this compound. EDP-305 is a non-BA FXR ligand, which contains steroidal and non-steroidal components without carboxylic acid group (Wang et al., 2018). Some studies have reported that EDP-305 reduced liver fibrosis progression in rat with bile duct ligation (Erstad et al., 2018), attenuated interstitial renal fibrosis in a mouse unilateral ureteral obstruction model (Li S. et al., 2019), and improved liver injury and fibrosis in mouse models of biliary and metabolic diseases (An et al., 2020).

### FXR Antagonists

#### Bile Acid Antagonists

Although BAs are considered as FXR agonists, some less hydrophobic BAs were still identified as FXR antagonists. Tauro-β-muricholic acid (T-β-MCA), a naturally occurring FXR antagonist, was reported to inhibit the FXR activation, which induced by taurocholic acid (TCA) or GW4064 (Sayin et al., 2013). More studies have demonstrated that T-β-MCA is an endogenous FXR antagonist in the intestine, which can inhibit basal FXR signaling and TCA-activated signaling (Li et al., 2013; Liu et al., 2020). Moreover, taurochenodeoxycholic acid (TCDCA), a weak agonist of FXR, can also inhibit the FXR activation by TCA and inhibits Shp mRNA expression (Li et al., 2013). Glycoursodeoxycholic acid (GUDCA) was another BAs, which was identified as an intestinal FXR antagonist showing effective therapeutic potency on obesity and type 2 diabetes (Cherney and Lam, 2018; Sun et al., 2018). It was also reported that glycine-β-muricholic acid (Gly-MCA) inhibited FXR signaling exclusively in intestine, resulting in the decrease of serum and intestine ceramide level and the improvement of metabolic dysfunction in obesity mouse (Jiang et al., 2015b).

#### Non-Bile Acid Antagonists

Guggulsterone (4,17(20)-Pregnadiene-3,16-dione) is a phytosterol found in the resin of the guggul plant, which inhibited CDCA-induced FXR activation in concentrations above 10 μM and was identified as FXR antagonists (Urizar et al., 2002). Guggulsterone has a significantly inhibitory effect on NF-kB signaling and attenuated the expression of proinflammatory cytokines, showing the therapeutic potential on Graves’ orbitopathy, colorectal cancer and atherosclerosis (Gautam et al., 2019; Leo et al., 2019; Kim et al., 2020). Epiallopregnanolone sulfate (PM5S) is closely associated with intrahepatic cholestasis of pregnancy (ICP) and elevates in the serum of ICP patients. PM5S can inhibited FXR activation resulting in the decrease of FXR-mediated bile acid efflux (Abu-Hayyeh et al., 2013). 3,5-disubstituted oxadiazole core (a new chemotype of FXR antagonists) (Festa et al., 2019), Stigmasterol (a Soy Lipid-Derived Phytosterol) (Carter et al., 2007), tuberatolides (isolated from the Korean marine tunicate Botryllus tuberatus) (Choi et al., 2011), andrographolide (representative active ingredient of Andrographis panniculata Nees) (Kumar et al., 2020) were also identified to have antagonistic activity on FXR. However, the effect and mechanism of FXR inhibition by these FXR antagonists also need further study and exact evidence.

## Role of FXR in Cardiovascular Diseases

Several studies reported that the relative risk for developing CVD is double that of the general population in patients suffering from cardiometabolic syndrome, accompanied with an increase in all-cause mortality (Ford, 2005; Galassi et al., 2006; Gami et al., 2007). At the beginning of the 21st century, CVD, including hypertension and atherosclerosis, were regarded as one of the main risk factors for developing cardiometabolic disease. China already has the largest absolute disease burden of hypertension in the world (Li et al., 2015). In 2010, approximately 265 million Chinese adults had hypertension. The development of essential hypertension is complex, particularly with obesity, glucose metabolism disorders, and inflammation involved. FXR has an extensive role in regulating cholesterol, lipid and glucose metabolism (Li and G, 2015; Massafra et al., 2018). And FXR activation has been shown to limit the inflammatory response (Massafra et al., 2018). It is widely known that disorders involving the secretion of endothelial factors play important roles in the pathogenesis of hypertension. The regulatory effect of FXR on the secretion of endothelial factors suggests it plays a key role in mediating the pathophysiological effects of hypertension. Indeed, recent studies have revealed novel functions of FXR in hypertension. For instance, it was reported that FXR activation upregulates endothelial nitric oxide synthase (eNOS) expression (Li et al., 2008) and down-regulates endothelin-1 (ET-1) expression (He et al., 2006) by negatively interfering with the activator protein 1 (AP-1) signaling pathway in cultured human vascular endothelial cells. FXR activation also inhibited vascular contraction and induced concentration-dependent relaxation in normal aorta through nitric oxide (NO) mechanism (Zhang et al., 2012). In addition, it was found that treatment with the FXR agonist CDCA decreased blood pressure in spontaneously hypertensive rats (SHRs) by improving vasorelaxation and reducing vasoconstriction (Lin, 2015). Attenuation of the inflammatory response in mesenteric resistant arteries by altering eNOS and ET-1 levels could be a possible mechanism. These findings support a potential role for FXR as a regulator in vascular activities and the treatment of hypertension.

Since FXR regulates the expression of genes critical for BA and lipid homeostasis, research has long been focused on the role played by FXR in the initiation and progression of atherosclerosis. Many studies were undertaken to investigate the pathological consequences of the loss of FXR function on the risk and severity of atherosclerosis. Hanniman et al. (2005) reported that Fxr^−/−^ApoE^−/−^ mice fed a high fat/high-cholesterol diet developed larger atherosclerotic lesions. This study on a mouse model of atherosclerotic disease suggested that loss of Fxr function is associated with decreased survival, increased severity of defects in lipid metabolism, and more extensive aortic plaque formation. However, two other studies found reduced atherosclerosis development in *Fxr*^−/−^*Apoe*^−/−^ mice and *Fxr*^−/−^ *Ldlr*^−/−^ mice (Guo et al., 2006; Zhang et al., 2006). In contrast, another study has reported that LDL receptor (*Ldlr*) knockout (KO) mice with Fxr and Tgr5 dual deficiency developed severe atherosclerosis and aortic inflammation through activation of the NF-κB signaling pathway (Miyazaki-Anzai et al., 2018). Due to inconsistent results from studies of FXR loss-of function models, the role of FXR in the development of atherosclerosis remains unclear. Despite these discrepancies, it is generally agreed that FXR activation is antiatherogenic. Many studies have shown that the synthetic FXR agonists, such as INT-767 (Jadhav et al., 2018; Miyazaki-Anzai et al., 2018), INT-747 (Mencarelli et al., 2009), WAY-362450 (Hartman et al., 2009), improved blood lipid and reduced aortic plaque formation. FXR agonists induced SHP expression and repressed cholesterol 7alpha-hydroxylase (CYP7A1) and sterol 12 alpha-hydroxylase (CYP8B1) expression (Hartman et al., 2009). FXR agonists were found to induce small heterodimer partner (SHP) expression and repress cholesterol 7alpha-hydroxylase (CYP7A1) and sterol 12 alpha-hydroxylase (CYP8B1) expression (Mencarelli et al., 2009). Interestingly, researcher also found that the effects of FXR agonist are different according to sex. Female but not male mice had reduced aortic lesion formation on treatment with the potent synthetic FXR agonist WAY-362450 (Hartman et al., 2009). FXR controls the expression of multiple genes that are key to many aspects of CVD. Li et al. (2019b) found that activation of FXR increased FNDC5 mRNA expression in human and increased the circulating level of irisin in Rhesus macaques and level of irisin is related to the degree of atherosclerosis in *Apoe*^−/−^ mice.

During heart failure, decreased vascular bioavailability of NO leads to the attenuation of coronary or systemic vasodilatation (Chen et al., 2002; Chen et al., 2003; Chen et al., 2005). Asymmetric dimethylarginine (ADMA) can limit NO bioavailability and increase production of eNOS derived reactive oxygen species. High plasma ADMA levels are considered as an increased risk for cardiac death (Boger et al., 2009). Dimethylarginine dimethylaminohydrolase 1 (DDAH1) can cause ADMA degradation. DDAH1 plays an important role in regulating the cardiovascular function and risk factors of congestive heart failure (CHF) by maintaining cardiovascular NO/cGMP/PKG signaling. Current, research shows that the FXR agonist GW4064 increases DDAH1 expression in the liver and kidney and decreases plasma ADMA (Hu et al., 2006; Li et al., 2009). These findings indicate that activating FXR to increase DDAH1 activity could be a promising strategy for improving NO bioavailability and maintaining cardiovascular function in the failing heart. However, it is unclear whether FXR activation induces DDAH1 expression in the cardiovascular system.

## Role of FXR in NAFLD and Cholesterol Metabolism

### NAFLD Is Associated With an Increased Risk of CVD

NAFLD is becoming the most common liver disease worldwide. In the US, approximately 25% of adults suffer from fatty liver without excessive drinking. In China, the incidence of fatty liver is increasing at a rate of 0.594% per year and it is anticipated that 20% of people in China will suffer from fatty liver by 2020 (Zhu et al., 2015). NAFLD includes a histological spectrum of conditions ranging from simple steatosis (SS) to steatosis plus necroinflammation (NASH). NAFLD is frequently associated with an increased risk of CVD and metabolic abnormalities, including obesity, diabetes, insulin resistance, hypertension, dyslipidemia, and atherosclerosis (Adams et al., 2017). A recent meta-analysis involving more than 8.5 million people in 22 countries showed that more than 80% of NASH patients were overweight or obese, 72% had dyslipidemia, and 44% had T2D, suggesting that NASH increased the incidence of CVD-related risk factors and metabolic diseases (Younossi et al., 2016). Further research has also confirmed that NAFLD is closely related to impaired left ventricular diastolic function, reduced myocardial energy metabolism and decreased coronary blood flow in patients with T2D (Rijzewijk et al., 2010; Mantovani et al., 2015). A clinical study suggested that NAFLD was independently associated independently with CVD, after adjusting for major demographic, clinical, and metabolic confounders (odds ratio, 1.23; 95% confidence interval, 1.04–1.44) (Stepanova and Younossi, 2012). This finding is further supported by a meta-analysis revealing that NAFLD is associated with an increased risk of CVD, hypertension, and atherosclerosis (Wu et al., 2016).

### Cholesterol Metabolism Disorder Plays a Key Role in NAFLD

Since NASH is closely related to the incidence of cardiometabolic diseases, they share many of the same pathological damage factors, including dyslipidaemia, oxidative stress, insulin resistance, inflammation, and endoplasmic reticulum (ER) stress (Lim et al., 2019). It should be noted that cholesterol metabolism disorder plays a key role in the pathological process of NAFLD, and a severe disturbance of cholesterol homeostasis in the cell leads to the accumulation of cholesterol and eventually cholesterol toxicity (Musso et al., 2010). It was reported that cholesterol accumulation or lipotoxicity induced oxidative injury, mitochondrial dysfunction, ER stress and inflammasome activation, which resulted in hepatocyte damage and liver fibrosis progression (Hager et al., 2012; Gan et al., 2014; Mridha et al., 2017). Dysregulation of cellular cholesterol homeostasis by increased activity of nuclear transcription factor-sterol regulatory binding protein 2 (SREBP2) and liver X receptor (LXR), and reduced activity of FXR plays a crucial role in in the accumulation of liver cholesterol in NASH (Musso et al., 2013).

### Effects of FXR Activation on NAFLD and Cholesterol Metabolism

FXR activation mediates BA metabolism and cholesterol homeostasis. FXR activation could lead to inhibition of the expression of the *CYP7A1* gene (encoding the rate-limiting enzyme in BA biosynthesis) by activating fibroblast growth factor 15 (FGF15) (Inagaki et al., 2005) or SHP (De Fabiani et al., 2001; Lee et al., 2018), contributing to BA metabolism and cholesterol homeostasis. A recent clinical study reported that variations in the *CYP7A1* gene are associated with elevated low-density lipoprotein cholesterol levels, and with increased risk of myocardial infarction and symptomatic gallstone disease (Qayyum et al., 2018). Therefore, the FXR/CYP7A1 pathway could be consider as a therapeutic target for cardiometabolic disease. Since cholesterol metabolism is closely related to the occurrence of NASH and CVD, more and more studies are focusing on the therapeutic effect of FXR agonists on NASH. Numerous clinical studies have demonstrated that OCA (a FXR agonist) has a significant therapeutic effect on NASH by increasing insulin sensitivity and reducing markers of liver inflammation and fibrosis (Mudaliar et al., 2013; Neuschwander-Tetri et al., 2015; Younossi et al., 2019). Clinical research also has revealed that cilofexor significantly reduced hepatic steatosis, liver biochemistry, and serum BAs (Patel et al., 2020). Additionally, animal studies have also shown that INT-767 reduced the severity of steatohepatitis, inflammatory infiltrates and fibrosis by restoring insulin sensitivity and promoting visceral fat brown adipogenesis and mitochondrial function (Comeglio et al., 2018; Roth et al., 2018). Tropifexor was reported to markedly prevent liver steatohepatitis and fibrosis by reducing oxidative stress and inflammatory injury (Hernandez et al., 2019). Overall, FXR activation has effective therapeutic effect on NAFLD and cholesterol metabolism, which may become a breakthrough for FXR agonists to be used in the treatment of cardiometabolic diseases.

## Role of FXR in High-Fat Dietary Consumption and Obesity

With the development of the social economy, the diet and lifestyle habits have changed drastically. The sharp increase in obesity rates worldwide poses a constant health challenge, which is associated with the morbidity and mortality of multiple diseases. At present, obesity is a leading risk factor for cardiometabolic diseases in the general population. Overweight and obesity lead to adverse metabolic effects on blood pressure, blood lipid and insulin resistance as well as increased occurrence of metabolic syndrome (Riobo Servan, 2013). Population-based epidemiological studies investigated the association of cardiometabolic risk factors with obesity indices, which can be used to aid screening for cardiometabolic risks in different population settings. A study of older adults in Colombia showed that the body roundness index (BRI) and waist-to-height ratio (WtHR), which are both obesity-related parameters, have a moderate discriminating power for detecting high cardiometabolic risks and are useful screening tools (Ramirez-Velez et al., 2019). Another study investigated the association of body mass index (BMI), waist circumference (WC) and body fat per cent (BF%) with cardiometabolic risk factors in Unguja Island, Zanzibar (Nyangasa et al., 2019). This study found that high BMI, WC and BF% were strongly associated with hypertension, with individuals with high WC being twice more likely to have hypertension. In the past, the importance of obesity as a risk factor for cardiometabolic diseases has been underestimated among children and adolescents. A recent study performed a cross-sectional analysis of data from overweight or obese children and young adults 3 to 19 years of age to assess the prevalence of multiple cardiometabolic risk factors according to the severity of obesity (Skinner et al., 2015). They found that severe obesity in children and young adults was associated with an increased prevalence of cardiometabolic risk factors, particularly among boys and young men. Accordingly, there is a need for effective interventions to create awareness as well as for primary prevention public health strategies aimed at preventing cardiometabolic diseases caused by obesity.

The FXR is an important regulator in the promotion of lipid metabolism and organismal energy metabolism, as well as in reducing inflammation. Based on these findings, the specific targeting of FXR may be an effective approach to treat obesity-induced cardiometabolic diseases. A central role of FXR in lipid homeostasis has been confirmed in mice with gene ablation or HFD-induced obesity. High-fat diet-fed intestine-specific Fxr-null mice were less obese compared with their wild-type counterparts (Li et al., 2013). Mice with intestine-specific FXR disruption had reduced hepatic triglyceride accumulation in response to a HFD-induced (Jiang et al., 2015a). Meanwhile, researcher also found that sex specific expression of lipid-related genes, including Fas, Colla1, Timp1, and Smpd3, may be FXR-dependent (Jiang et al., 2015b). However, other studies have found that lack of FXR can adversely affect lipid metabolism. Fxr/Bar null mice and Fxr-deficient mice have increased hepatic cholesterol, triglycerides and high-density lipoprotein cholesterol, as well as a proatherogenic serum lipoprotein profile (Sinal et al., 2000; Lambert et al., 2003). And there is no gender-based difference in this adverse effect. FXR knockout mice do not display sex-specific expression of lipid- and bile acid-associated genes (Sheng et al., 2017). Lipid metabolism can be increased when FXR is activated. When CDCA is given to hamsters fed a HFD, it significantly reduces triglycerides and very-low-density cholesterol (Bilz et al., 2006). They also found that activation of FXR facilitates the clearance of very-low-density cholesterol and chylomicrons by repressing the expression of microsomal triglyceride transfer protein and apolipoprotein B (Zhang et al., 2004).

Despite recent advances in understanding the relationship between FXRs and obesity, the specific mechanism by which FXR regulates obesity requires further studies. Some studies analyzed the hepatic genome-wide binding sites of FXR in healthy mice and mice with diet-induced obesity. They found that more FXR-binding sites are likely functionally inactive in obesity and direct gene repression by agonist-activated FXR is common (Lee et al., 2012). In addition, the beneficial effect of bariatric surgical procedures, such as vertical sleeve gastrectomy (VSG), may also be related to FXR. Since FXR can maintain weight loss after VSG, it is considered as an important molecular underpinning for the beneficial effects of this weight-loss surgical procedure (Ryan et al., 2014). Although the different research studies reach different or even contrary conclusions, they suggest that inhibition of intestinal FXR is a potential target for anti-obesity drug development.

## Role of FXR in Insulin Resistance and Diabetes Mellitus

Insulin resistance refers to a state of weakened insulin response, which is a common feature of T2D, obesity, and hypertension (Rask-Madsen and Kahn, 2012). T2D is associated with chronic inflammation, characterized by the release of excessive proinflammatory cytokines, acute-phase proteins and other mediators, all of which are important damage factors for CVD (Paneni et al., 2013). Insulin resistance is not only an important pathological factor of diabetes, but is also considered as the underlying cause of the development of the cardiometabolic syndrome. It has been reported that fasting insulin (an indicator of insulin resistance) contributed to the development of the metabolic syndrome, including hypertension, hypertriglyceridemia, reduced HDL-C, and T2D (Haffner et al., 1992). Insulin resistance is the direct cause of elevated fasting glucose and prediabetes (impaired glucose tolerance) contributing to glycotoxicity, lipotoxicity, and inflammation, all of which trigger and accelerate vascular damage, endothelial dysfunction, hypertension, atherosclerosis and CVD (Ouchi et al., 2011). There are many factors that cause insulin resistance, including dysfunctional binding to insulin receptor, abnormal insulin secretion, lipid oversupply and alterations in substrate metabolism (Roberts et al., 2013). Elevated plasma-free fatty acids played a key role in the development of insulin resistance and T2D, and the decrease in plasma-free fatty acids was closely correlated with improvement in insulin sensitivity in T2D (r=0.76) subjects (P< 0.001) (Daniele et al., 2014). Excess free fatty acid can simultaneously cause insulin resistance, activate oxidative stress and ER stress, ultimately leading to the secretion of a variety of proinflammatory cytokines, both of which could conversely produce the development of insulin resistance (Boden, 2011). As the key regulatory element of free fatty acid metabolism, FXR has effective therapeutic effect on insulin resistance and T2D.

A recent study in a rabbit model of HFD-induced metabolic syndrome indicated that long-term treatment with INT-767 decreased HFD-induced fatty acid synthesis and fibrosis, while increasing lipid handling and improving insulin resistance (Comeglio et al., 2018). Insulin resistance is associated with increased pro-inflammatory cytokines and decreased anti-inflammatory cytokines released by adipose tissue. FXR activation was found to increase the release of anti-inflammatory cytokines and insulin-sensitive adipokines (adiponectin and leptin), and then reversed insulin resistance (Shihabudeen et al., 2015). FXR agonist fexaramine (Fex) was reported to active white adipose tissue and reduce insulin resistance enhancing glucose tolerance and lowering inflammatory cytokine levels (Fang et al., 2015). FXR activation reduced liver expression of genes involved in fatty acid synthesis, lipogenesis, and gluconeogenesis, thereby reversing the development of insulin resistance and liver steatosis in *fa/fa* rats (Cipriani et al., 2010). A clinical study also has reported that administration of 25 or 50 mg of OCA for 6 weeks increased insulin sensitivity in patients with T2DM and NAFLD (Mudaliar et al., 2013). Recent studies indicate that FXR has the potential to be a therapeutic target for T2D. HS218, a new FXR specific antagonist, suppressed liver gluconeogenesis and effectively improved glucose homeostasis in db/db and HFD-induced T2D mice by inhibiting Fxr binding to PGC-1α promoter (Xu et al., 2018). FXR activation mitigated tacrolimus-induced DM by regulating gluconeogenesis as well as glucose uptake of renal in a PGC1α/Foxo1-dependent manner (Li L. et al., 2019). In summary, FXR has a significant regulatory effect on insulin resistance and T2D. However, further study is still needed to confirm the therapeutic potency and mechanism of FXR agonists on T2D.

## Role of FXR in Intestinal Microorganism

The human gut harbors more than 10 trillion microbial cells, which provide unique metabolic functions to the host. Commensal gut microbiota is said to act as a “signaling hub” in many pathophysiological functions of the mammalian host (Cani, 2018). The gut microbiome plays a critical role in host susceptibility to and risk of disease (Sender et al., 2016). Among them, the association between gut microbiota and cardiometabolic diseases has attracted wide attention. Numerous studies have shown that intestinal bacteria are closely related to cardiometabolic diseases. Differences in microbial richness, species abundance, and microbial community structure might be involved in the pathogenesis of cardiometabolic diseases. Patients with symptomatic atherosclerosis were found to have increased number of the genus *Collinsella*, while the healthy controls had an increased abundance of *Eubacterium* and *Roseburia* (Karlsson et al., 2012). *Candida*, *Campylobacter*, and *Shigella* species apparently increased with the duration and aggravation of the disease. The importance of gut microbiota in CVD development is evident in germ-free animal models. For example, Li et al. (2017) transferred feces from hypertensive patients to germ-free mice and found elevated blood pressure in germ-free mice exposed to feces. Also, Stepankova et al. (2010) found that germ-free Apoe^-/-^ mice fed the low-cholesterol standard diet had increased atherosclerotic plaques compared with their conventionally reared counterparts with defined microflora. However, it should be noted that other researchers have come to the opposite conclusion, and they suggested that gut microbiota could accelerate the formation of atherosclerosis (Kasahara et al., 2017). Although there is controversy about the precise molecular mechanisms by which gut microbiota influences cardiometabolic diseases, there is no doubt that targeting gut microbiota is an effective strategy for the treatment of cardiometabolic diseases. Numerous research studies have identified the gut microbiota as a novel regulator of cardiometabolic diseases. For instance, 12-week administration, *via* drinking water, of *E*. *coli* Nissle 1917 (pNAPE-EcN) expressing the endogenous lipid satiety factors N-acyl phosphatidylethanolamines (NAPEs), were found to improve various indices of cardiometabolic disease in Ldlr^-/-^ mice (May-Zhang et al., 2019).

FXR and gut microbiota are closely linked. For instance, FXR alters the gut microbiota composition, and transplantation of the gut microbiota into germ-free mice changes the lean phenotype of *Fxr* knockout donor mice, indicating that Fxr may contribute to increased adiposity by altering the microbiota composition (Schoeler and Caesar, 2019). The FXR-gut microbiota interaction plays an important role in most forms of CVD. Trimethylamine N-oxide (TMAO) is an intestinal bacterial-related metabolite. Trimethylamine (TMA), the precursor of TMAO, is oxidized by the host hepatic enzyme flavin monooxygenase 3 (FMO3) to generate TMAO (Bennett et al., 2013). High levels of TMAO represent a strong prognostic biomarker of cardiovascular events (Wang et al., 2011). The study found FXR could induce FMO3 expression, thereby increasing plasma TMAO levels (Bennett et al., 2013).

About 95% of BAs are reabsorbed and transported back to the liver *via* the portal circulation, but the remaining 5% will be transformed into secondary BAs by intestinal microorganisms by microorganisms in the intestine. BA metabolism is modulated by BA biotransformation in the intestine. Gut microbiota-derived secondary BAs play important roles in the development of atherosclerosis through the modulation of FXR. Sayin et al. (2013) found that gut microbiota regulated expression of FGF15 in the ileum and CYP7A1 in the liver through FXR-dependent mechanisms so as to maintain the balance of cholesterol metabolism. Sun et al. (2018) found that *Bacteroides fragilis* was decreased and the BA glycoursodeoxycholic acid (GUDCA) was increased in the gut of patients with T2D after metformin treatment. These changes were accompanied by inhibition of intestinal FXR signaling, which indicated that metformin acts in part through a *Bacteroides fragilis*–GUDCA–intestinal FXR axis to improve metabolic dysfunction. These findings also suggest that microbiota-derived secondary BAs might be involved in signaling pathways that regulate lipid and glucose metabolism due to their binding affinity to FXR thereby playing a role in the regulation of cardiometabolic disease and vascular function. In addition, some studies suggest that there is sex-specific interplay among the gut microbiome, FXR, and BAs. Women reportedly produce higher concentrations of secondary BAs compared to men (Baars et al., 2018), therefore, perhaps, women harbor more gut microbiota that are capable of bile acid transformations. Secondary bile acids may then activate a number of downstream targets, including FXR, having potential mixed effects on CVD risk (Claudel et al., 2005).

## Role of FXR on Aging

Aging is an inevitable natural process and also a complex process that takes place in all living organisms. At present, there is a growing population of elderly people. Aging population is a big challenge and a great economic burden all over the world. It is expected that the population over 65 years of age will reach 22% by 2040 (Heidenreich et al., 2011). The high incidence rate of age-related degenerative diseases brings impact to the social health system. Now people have realized that aging is an important risk factor in the development of CVD (North and Sinclair, 2012). To effectively halt the progression of cardiometabolic disease, researcher actively explore the molecular mechanisms behind declined organs function in aging. Inflammation (Cevenini et al., 2010) and cellular oxidative stress (Chen et al., 2007) are considered as key mechanisms that aging induced diseases. The inflammatory biomarkers related to aging, such as high-sensitivity C-reactive protein (hs-CRP), IL6 and TNF, presenting high circulating concentrations (Singh and Newman, 2011), leading to a chronic overproduction of ROS (Dinh et al., 2014) and mitochondrial dysfunction later in life (Anderson et al., 2018). These factors can lead to arterial dysfunction. And the main changes are increased stiffness of the large arteries and reduced vascular endothelial function (Lakatta and Levy, 2003). The vascular endothelial function dysfunction can aggravate inflammation and oxidative stress, forming a vicious circle. These pathological changes induced by aging which partially explains the pathogenesis of cardiometabolic disease include hypertension, atherosclerosis and T2D. In addition, it should be noted that aging presents a unique challenge to the prediction of cardiometabolic risk in men and women as both sex hormone-dependent and sex hormone-independent effects play various roles in the development of aging related cardiovascular diseases in men versus women (Faulkner and Belin de Chantemele, 2019). Therefore, a better understanding mechanism of aging contributes to the formulation of new therapeutic strategies for age-related cardiovascular diseases and improvement the quality of life.

As aging progresses, chronic imbalance of energy intake and expenditure promote hepatic steatosis and muscle insulin resistance (Shulman, 2014). Many studies have shown increased body weight and decreased physical activity resulting in glucose intolerance and insulin resistance in aging mice (Kenyon, 2010; Houtkooper et al., 2011). FXR and SHP selectively expressed in liver and have pivotal functions in metabolic pathways such as bile acid homeostasis, fatty acid and glucose metabolism. Previous study has shown that FXR reduction was responsible for the hepatic TG accumulation in aging mice, suggesting that FXR dysfunction might be involved in the development of hepatosteatosis in aging mice (Xiong et al., 2014). On the other hand, research has found that combined deletion of the hepatic FXR/SHP axis improves glucose/fatty acid homeostasis in aged mice, reversing the aging phenotype of body weight gain, increased adiposity and glucose/insulin tolerance (Kim et al., 2017). Moreover, FXR-deficient C57BL/6 mice were more prone to spontaneous development of NASH than wild-type upon aging (Bjursell et al., 2013).

## Conclusion

**Figure 1:**
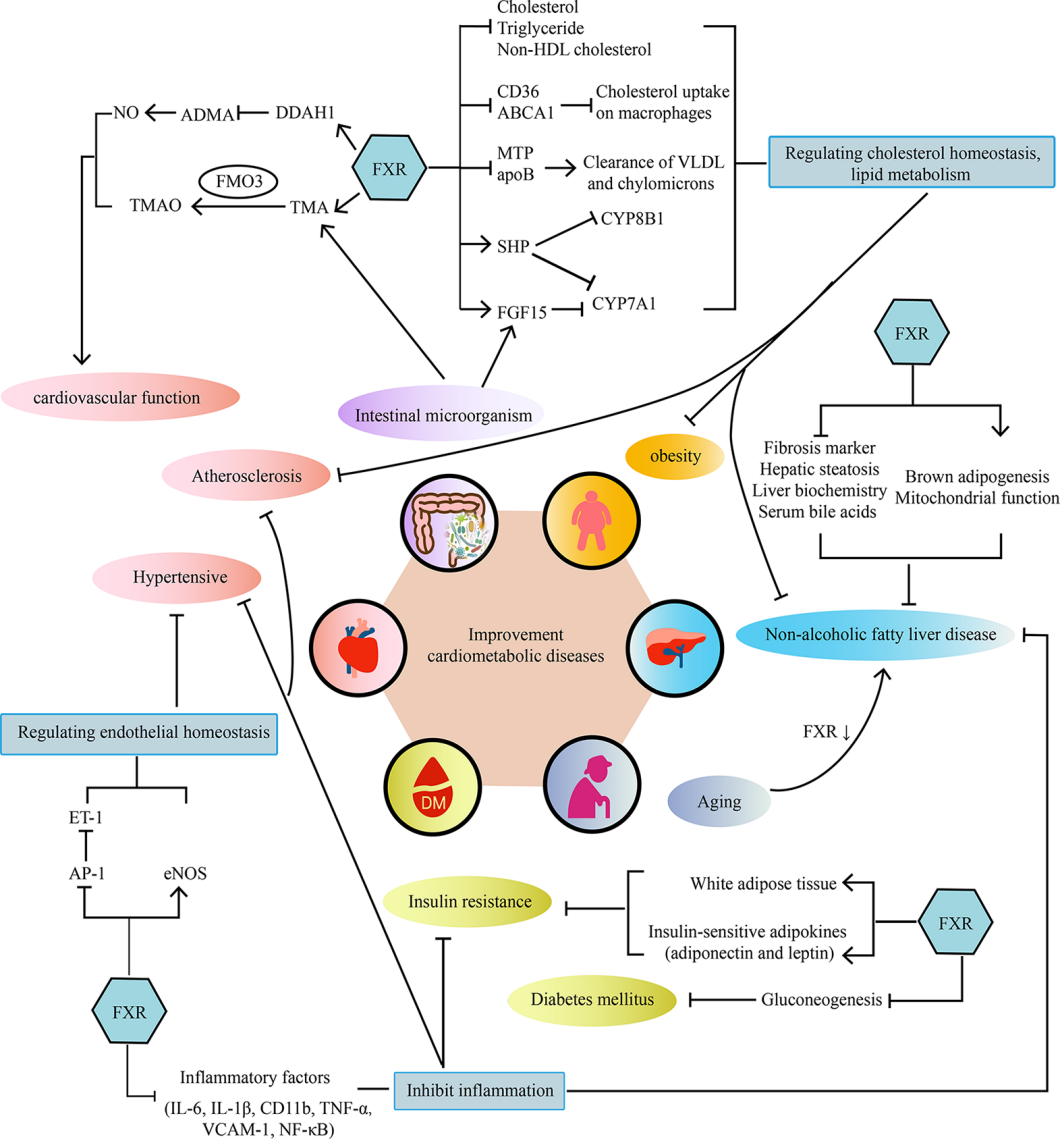
The main mechanisms of the effect of FXR on cardiometabolic diseases. FXR acts on cardiometabolic diseases in a multi-organ and multifactorial manner.

Cardiometabolic diseases are very complex diseases, which not only involve cardiovascular diseases, diabetes, and obesity, but also have complicated pathological mechanisms, including disorders of lipid metabolism, insulin resistance, abnormal glucose metabolism, oxidative stress, and inflammatory response. Intestinal microorganism also participates in the pathological process of cardiovascular disease through microbial metabolism. As the bile acids receptor, FXR plays a crucial role in bile acids metabolism and cholesterol homeostasis. Activating FXR could regulate lipid metabolism, maintain cardiovascular function, reduce insulin resistance, enhance glucose tolerance and decrease inflammatory cytokine levels. Therefore, more and more attention has been paid to the potential role of FXR as a regulatory factor in repairing damaged vessel, increasing insulin sensitivity and reducing markers of liver inflammation and so on. (As shown in **Figure 1**). Therefore, FXR agonists have the potential to become a new treatment strategy for cardiometabolic diseases. However, although FXR agonists have shown good clinical efficacy in the treatment of NAFLD/NASH, there is no definite clinical trial to confirm its benefit for cardiovascular disease. Further well-designed clinical trials of FXR agonists are required to prove their efficacy in cardiometabolic diseases and explore the underlying mechanism.

## Author Contributions

CL, JY, and WY searched for articles and wrote the paper. YW compiled the table. YQ drew the figure. YL proposed the topics and checked the whole manuscript. All authors contributed to the article and approved the submitted version.

## Funding

This work received support by National Nature Science Foundation of China (81774242, 81804006), China Postdoctoral Science Foundation (2020M672125), the Major Science Natural Science Foundation of the Shandong Province (ZR2018ZC1157), and Taishan Scholar Post Construction Fund (ts201712042).

## Conflict of Interest

The authors declare that the research was conducted in the absence of any commercial or financial relationships that could be construed as a potential conflict of interest.
